# Anterior deltoid muscle tension quantified with shear wave ultrasound elastography correlates with pain level after reverse shoulder arthroplasty

**DOI:** 10.1007/s00590-021-02987-1

**Published:** 2021-04-21

**Authors:** Jonas Schmalzl, Annabel Fenwick, Thomas Reichel, Benedikt Schmitz, Martin Jordan, Rainer Meffert, Piet Plumhoff, Dirk Boehm, Fabian Gilbert

**Affiliations:** 1 Department of Trauma, Hand, Plastic and Reconstructive Surgery, University Hospital of Wuerzburg, Oberduerrbacher Str. 6, 97080 Wuerzburg, Germany; 2 Department of Trauma, Orthopedic, Hand – and Plastic Surgery, University Hospital of Augsburg, Stenglinstrasse 2, 86156 Augsburg, Germany; 3 Orthopedic Surgery, König Ludwig Haus, University of Wuerzburg, Brettreichstrasse 11, 97074 Wuerzburg, Germany; 4 Hoechberg Orthopaeden, Hauptstraße 78, 97204 Hoechberg, Germany; 5 Ortho Mainfranken Wuerzburg, Bismarckstraße 16, 97080 Wuerzburg, Germany; 6 Department of Traumatology, Hand, Plastic and Reconstructive Surgery, University Hospital Wuerzburg, Josef-Schneider-Str. 2, 97080 Wuerzburg, Germany

**Keywords:** Shear wave elastography, Strain elastography, Shoulder, Deltoid muscle, Reverse shoulder arthroplasty; pain

## Abstract

**Introduction:**

Reverse shoulder arthroplasty (RSA) leads to medialization and distalization of the centre of rotation of the shoulder joint resulting in lengthening of the deltoid muscle. Shear wave ultrasound elastography (SWE) is a reliable method for quantifying tissue stiffness. The purpose of this study was to analyse if deltoid muscle tension after RSA correlates with the patients’ pain level. We hypothesized that higher deltoid muscle tension would be associated with increased pain.

**Material and methods:**

Eighteen patients treated with RSA were included. Constant score (CS) and pain level on the visual analogue scale (VAS) were analysed and SWE was performed on both shoulders. All three regions of the deltoid muscle were examined in resting position and under standardized isometric loading.

**Results:**

Average patient age was 76 (range 64–84) years and average follow-up was 15 months (range 4–48). The average CS was 66 points (range 35–89) and the average pain level on the VAS was 1.8 (range 0.5–4.7). SWE revealed statistically significant higher muscle tension in the anterior and middle deltoid muscle region in patients after RSA compared to the contralateral non-operated side. There was a statistically significant correlation between pain level and anterior deltoid muscle tension.

**Conclusion:**

SWE revealed increased tension in the anterior and middle portion of the deltoid muscle after RSA in a clinical setting. Increased tension of the anterior deltoid muscle portion significantly correlated with an increased pain level. SWE is a powerful, cost-effective, quick, dynamic, non-invasive, and radiation-free imaging technique to evaluate tissue elasticity in the shoulder with a wide range of applications.

**Level of evidence:**

Diagnostic study, Level III.

## Introduction

Reverse shoulder arthroplasty (RSA) changes the biomechanical properties of the shoulder joint and leads to distalization and medialization of the centre of rotation. This results in lengthening of the deltoid muscle of 10–20% and an increased deltoid wrapping angle [1]. On the one hand, an increased deltoid tension is crucial for a satisfying function after RSA, assuring stability and adequate range of motion. On the other hand, over-tensioning might lead to several problems such as acromial stress fractures, pain, and axillary nerve damage [1–3].

Ultrasound elastography is a well evaluated method for tissue evaluation of liver, thyroid or breast diseases [4–6]. Recently it has also been applied for the musculoskeletal system [7–10]. In this context, it has been shown to be a reliable method for detecting soft tissue properties and their changes caused by different conditions or pathologies. There are two main varieties—strain und shear wave elastography (SWE). Being a quantitative elastography method, SWE has been shown to be less examiner dependent than strain elastography. In addition, it is easily accessible, low-priced and there are no contraindications [11]. Tissue stiffness is calculated after application of an acoustic impulse (acoustic radiation force impulse, ARFI), which deforms the underlying tissue and induces tissue oscillation. These so-called shear-waves move transversally to the direction of the ultrasonic waves. The shear wave velocity depends on the elasticity of the examined tissue and is measured in metres per second (m/s). The measured shear wave velocity is converted to an elastic modulus using a mathematical equation [12]. To create a reliable result the probe has to be positioned parallel to the muscle fibres. In contrast to strain elastography, which represents a semi-quantitative method, there is no need for tissue compression during the exam. SWE is therefore able to provide quantitative information on the elastic modulus of the examined tissues. [13] The shoulder girdle including the rotator cuff and the deltoid muscle already have been subject to investigations [14–18]. Hatta et al. showed in an experimental design on 8 fresh frozen cadaver shoulders that EUS could be a reliable and feasible method to quantitatively assess the mechanical properties of the deltoid muscle by comparing elongated and native deltoid muscles [18]. However, not only the deltoid muscle but also the rotator cuff and the posterior shoulder capsule can be assessed as reported by Takenaga et al. and Muraki et al. [16, 19].

The purpose of this study was to use SWE to measure differences in deltoid muscle tension after RSA in different stages of muscle contraction and different portions of the deltoid muscle compared to the non-operated contralateral side. We hypothesized that deltoid muscle tension would be increased after RSA in rest and under isometric loading compared to the contralateral side and that higher deltoid muscle tension would be associated with an increased pain level.

## Materials and methods

### Study design

Eighteen patients treated with RSA could be included in this study. Surgery was performed by 3 senior consultants specialized in shoulder surgery. The surgical procedure was performed via the deltopectoral approach in all cases. A statistical power analysis was performed using G*Power 3.1 [20]. The minimal number of patients was set to be *n* = 16 (*β* > 0.8).

### Compliance with ethical standards

Institutional review board approval was obtained prior to commencing the study. All patients signed informed consent and gave their approval for the use of clinical and radiographic data for scientific purposes. The conducted experiments respect the ethical standards in the Helsinki Declaration of 1975, as revised in 2000, as well as the national law.

### Postoperative evaluation

Data concerning characteristics of the patient at the moment of surgery, surgical technique, and complications were retrospectively retrieved from our institution’s electronic medical record system.

An independent observer examined all patients and assessed the clinical outcome. For follow-up examination, the patients were asked to grade pain on a visual analogue scale (VAS). Active range of motion (ROM) was measured with a goniometer for elevation, abduction, and external rotation of the elbow at the side. Internal rotation was judged by the level of vertebra reached by the thumb. Functional outcome was assessed using the Constant-Murley score (CS).

### SWE examination

EUS was performed with Aixplorer (SuperSonic Imagine, Aix-en-Provence, France) using a 9 MHz linear array transducer. Conditions were identical for all examinations. The ultrasound examination was performed by one investigator (PP).

**Fig. 1:**
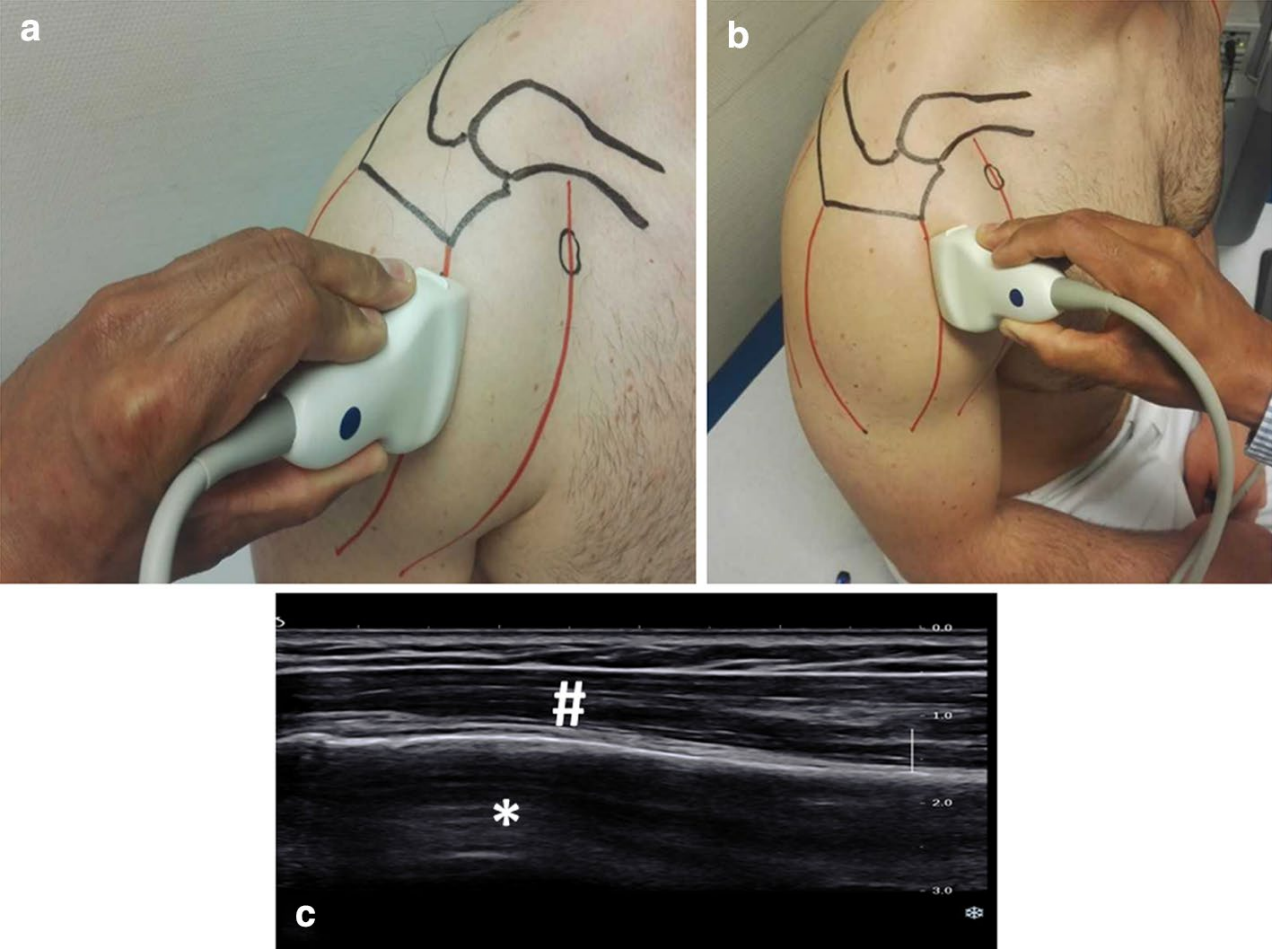
Examination set-up. Standardized examination set-up with the patient in a sitting position with the arm placed palm down on the patient’s thigh (**a**, **b**). Exemplary ultrasound image (**c**) showing the humeral neck (*) and the deltoid muscle (#)

The three anatomic regions of the deltoid muscle (anterior, middle and posterior portion) were separately examined and measured with parallel alignment of the ultrasound probe to the muscle fibres [18]. As reference for the probe position the humeral neck was visualized as shown in Fig. 1. Each region of interest of the deltoid muscle on both sides was examined in a resting position and under maximum isometric contraction in 60° abduction using a flexibar (IsoForceControl® Ca. Medical Device Solutions AG, Oberburg, Switzerland). To standardize the resting position, the patients were seated and the arm was placed palm downwards on the thigh as shown in Fig. 1.

Instead of using various measurement points, the specific deltoid area of interest was encircled manually without interference by fascia or bone and shear wave velocity as well as the elasticity modulus as reference for tissue stiffness were calculated. Tissue oscillation reaches an amplitude of up to 20 µm, and shear wave velocity depends on the elasticity of the examined tissue, ranging from 1 to 10 m/s. In a time range of 5 ms, the moving tissue returns to its initial position [21]. By tracking the beams of the transducer, the shear wave propagation velocity can be traced. SWE therefore provides quantitative information on the elastic modulus of the examined tissues using an algorithm.

### Statistical analysis

Statistical analysis was performed using SPSS version 22 (IBM, Armonk NY, USA). Parameters were tested for normal distribution and the level of significance calculated for dependent samples by Mann Whitney U test and Kruskal Wallis test. Pearson’s correlation was used to evaluate a relation between pain level, clinical outcome and tissue properties. Differences were considered statistically significant if *p* < 0.05.

## Results

Mean patient age was 76 (range 64–84) years. The follow-up examination was carried out on average 15 months postoperatively (range 4–48). Patients’ baseline characteristics are illustrated in Table 1.

**Table 1:** Baseline characteristics

Variable	Number
Mean patient age in years [range]	76 [64–84]
Mean follow-up in months [range]	15 [4–48]
*Gender*	
Male [percent]	5 [28%]
Female [percent]	13 [72%]
*Side*	
Right [percent]	12 [67%]
Left [percent]	6 [33%]
*Indication*	
Cuff tear arthropathy [percent]	12 [67%]
Proximal humeral fracture [percent]	6 [33%]

Mean postoperative active forward flexion was 150°, mean abduction 140° and mean external rotation at the side was 25°. Mean internal rotation was at lumbar vertebra 5. The mean CS was 66 points (range 35–89). Average pain level on the VAS was 1.8 out of 10 points (range 0.5–4.7).

**Fig. 2:**
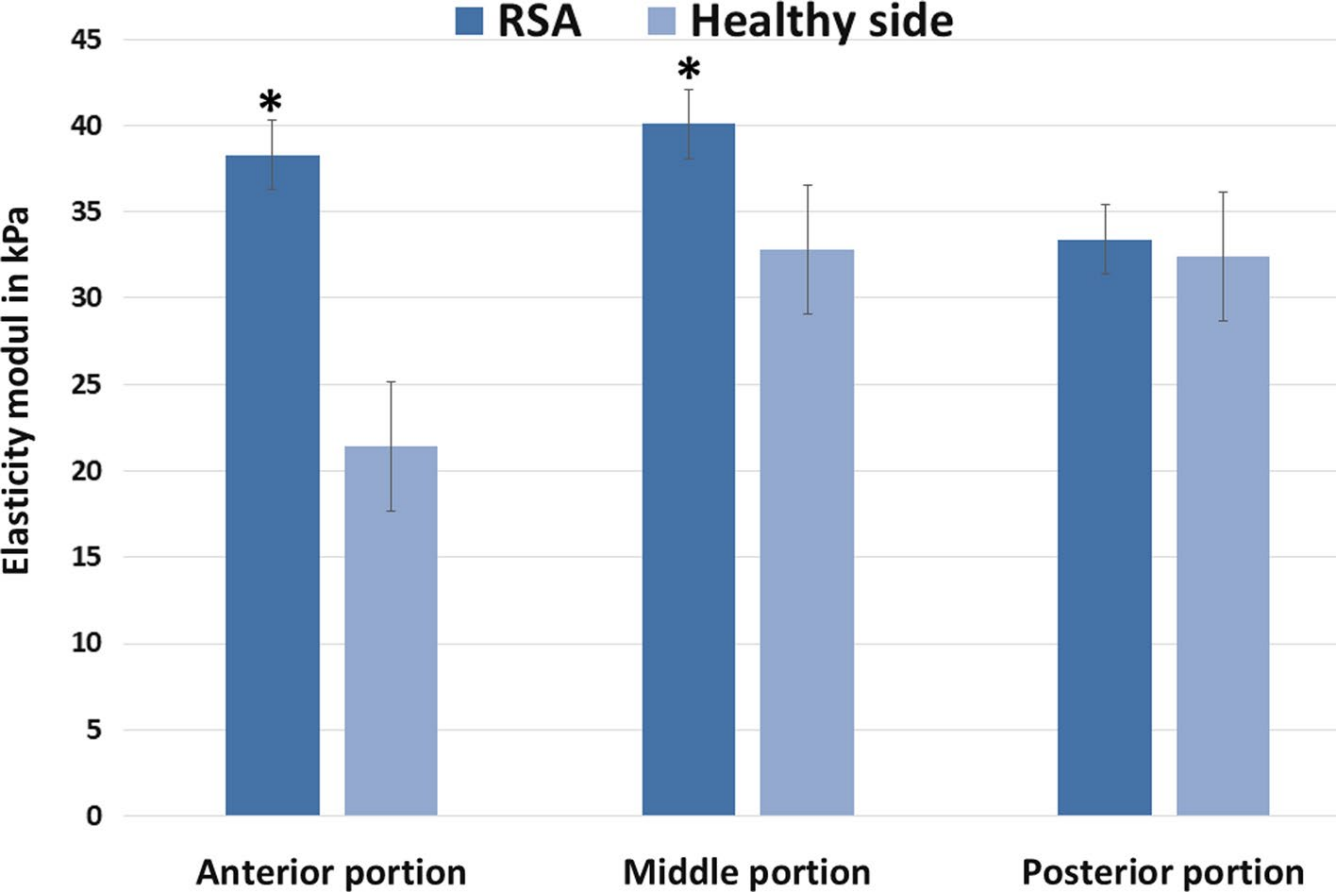
Muscle elasticity in resting position. Shear wave elastography shows a change of deltoid elasticity in each region of the deltoid with an increased tension after RSA than on the healthy contralateral side especially in the anterior and middle portion of the deltoid in resting position without loading (*p* < 0.05). *RSA* reverse shoulder arthroplasty; *kPa* kilo Pascal

Under minimum muscle contraction in the resting position SWE showed higher muscle tension of the deltoid after RSA compared to the contralateral, non-operated side as shown in Fig. 2. The differences were statistically significant for the anterior and middle portion of the deltoid muscle.

**Fig. 3:**
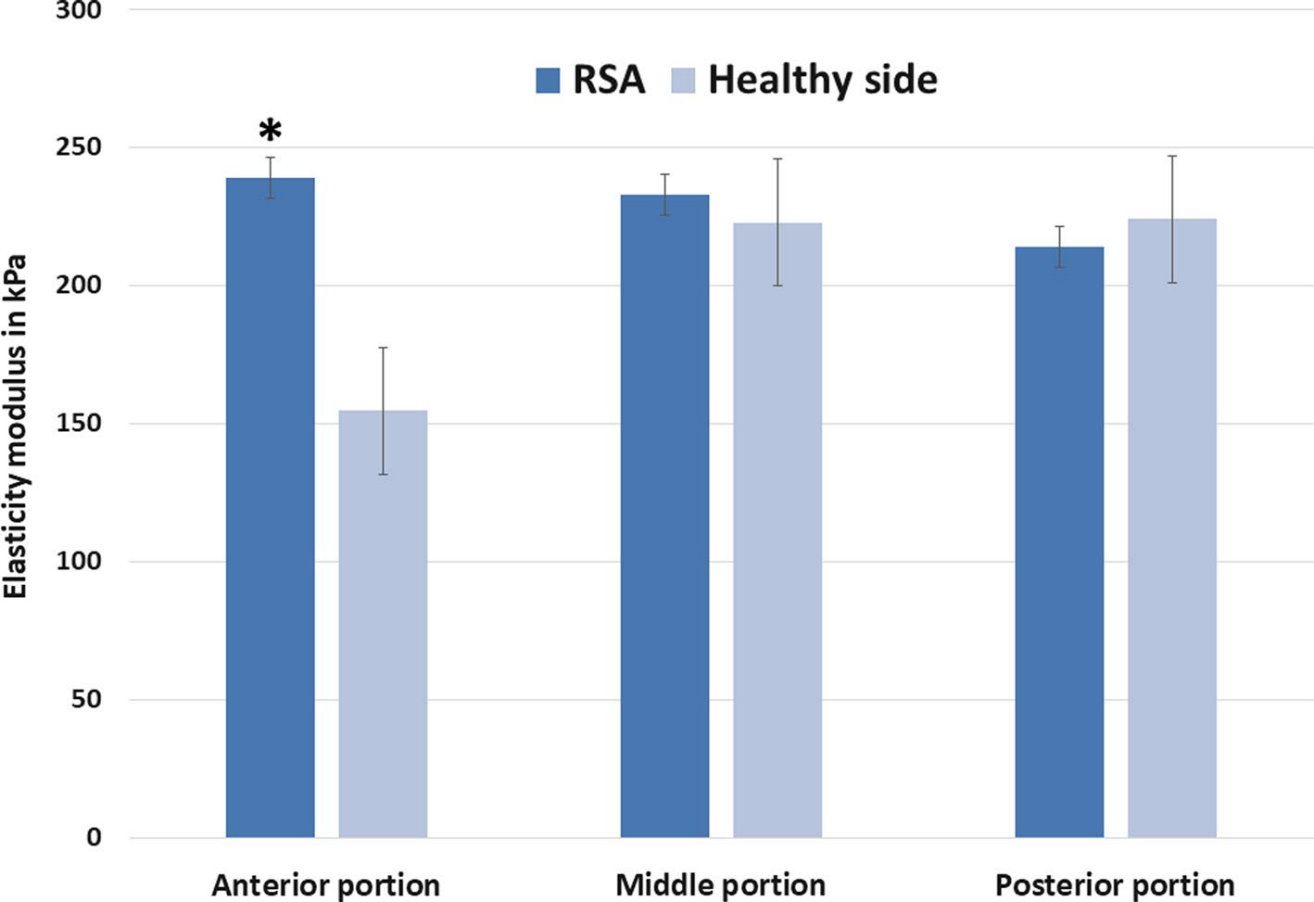
Muscle elasticity under isometric contraction. Isometric loading of the contralateral healthy side also leads to an increased tension of all areas of the deltoid muscle. Differences under isometric loading were only significant for the anterior portion

Under isometric loading a higher muscle tension was observed in the shoulders with RSA compared to the contralateral side only for the anterior portion (RSA: 239 kPa ± 160; contralateral side: 155 kPa ± 114; *p* < 0.05) (Fig. 3).

**Fig. 4:**
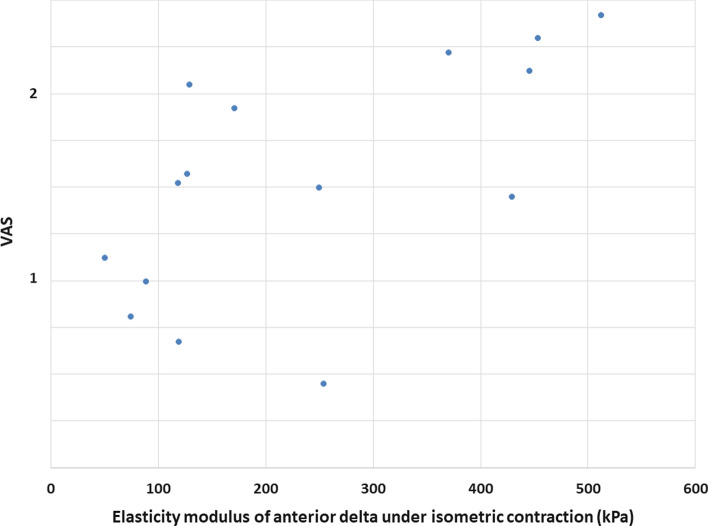
Muscle tension under isometric loading correlated with overall pain level. Scatter plot of the elasticity modulus of anterior portion of the deltoid muscle (*x*-axis) and the patients reported pain on the visual analogue scale (*y*-axis). *VAS* visual analogue scale

For the anterior portion we observed a statistically significant (*p* = 0.005) and strong correlation (Pearson’s correlation coefficient *R* = 0.63) between the muscle tension and patients’ overall pain level under isometric muscle contraction as shown in Fig. 4.

## Discussion

The changes of the biomechanical properties in the shoulder joint after RSA have been described extensively [22]. However, up to date it remains unclear how much deltoid muscle tissue tension is necessary for an optimal function of RSA.

In 1998 SWE was first described by Sarvazyan et al. as a reproducible ultrasound method for the quantification of tissue elasticity [21]. Later on, Kim et al. showed that SWE represents an excellent method for evaluating muscle stiffness in the shoulder with a high inter- and intraobserver reliability [23]. Therefore, we decided to assess deltoid muscle tension after RSA using SWE.

In this study we could show significantly higher muscle tension in the anterior and middle deltoid muscle region in patients after RSA compared to the contralateral non-operated side. In addition, we observed a strong correlation between the muscle tension and patients’ overall pain level under isometric muscle contraction.

Our study confirms the experimental findings by Hatta et al. in 8 fresh-frozen cadaver shoulders. They showed, that distalization of the humeral shaft led to increased muscle stiffness especially in the anterior and middle portion of the deltoid muscle.[18]

Roche et al. simulated deltoid lengthening in a computer model for different reversed shoulder protheses and for each type of motion [24]. They found that deltoid lengthening was higher in the anterior and middle portion than in the posterior portion, both in external and internal rotation. The simulated asymmetric lengthening of the deltoid muscle after RSA could now be confirmed through our in vivo measurements showing higher tension in the anterior and middles portion.

Schwartz et al. described in a cadaveric biomechanical study that the anterior portion is crucial for the function of RSA [25]. They found that abduction moment arm of the anterior and middle portion significantly increased after RSA.

To our knowledge this is the first study supporting these experimental findings in an in vivo setting.

Deltoid lengthening is recognized empirically as an important clinical attribute for the function of RSA. [26]

Since the publication of a study by Ascione et al. showing an increased prevalence of scapular spine fractures after RSA with an onlay-design (i.e. with additional lengthening of the arm due to a humeral onlay polyethylene component) everybody should be aware of the risks of overlengthening the arm [27].

In addition, Sabesan et al. showed a moderately negative correlation between deltoid lengthening and improvement in forward elevation, i.e. forward flexion was reduced in patients with excessive overlengthening [28].

Therefore, it is obvious that there is a limit to deltoid lengthening, a point where the tension is too high; however, up to date intraoperative adjustment of the optimal soft tissue tension remains highly subjective.

As mentioned above, in our study we could show that the tension of the deltoid muscle correlates strongly with the overall pain level reported by the patients. However, we could not show a negative influence on the functional outcome. This might be due to the small size of the study cohort or the underrepresentation of pain in the CS.

Thus, we could show that SWE represents a powerful tool to measure postoperative deltoid muscle tension and might help to define recommendations and cut-off values for analysing and adjusting the deltoid forces in RSA.

### Limitations

An important limitation of SWE is that the region of interest is placed manually. Thus, regional inhomogeneities may lead to incorrect values of overall shear wave propagation velocity.

In addition, the exact orientation of the transducer is essential for generating reproducible results. Experimental studies showed that SWE can measure muscle stiffness in the supraspinatus muscle and identify regions of different elasticity in the muscle. Nevertheless, it is fundamental to place the ultrasound probe parallel to the muscle fibres to achieve reliable results.

Moreover, muscle stiffness is strongly affected by the grade of muscle contraction. This is supported by the findings of Muraki et al., who published significant changes in muscle stiffness at different levels of muscle contraction in a cohort of 23 healthy individuals using SWE [15]. Therefore, it is highly recommended to measure muscle relaxation or to use at least a standardized position while performing SWE analysis. In our study we only used a standardized arm position for the measurement.

## Conclusion

In this study we could show that deltoid tension increases after RSA especially in the anterior and middle portion. These in vivo findings support recently published experimental studies. Increased tension of the anterior deltoid muscle portion significantly correlated with an increased pain level. SWE could potentially be used to identify patients at risk for an acromial stress fracture or malfunction of RSA due to overlengthening. Further possible applications might be the intra- and postoperative assessment of ideal deltoid tension to optimize results after RSA. SWE is a powerful, cost-effective, quick, dynamic, non-invasive, and radiation-free imaging technique to evaluate tissue elasticity in the shoulder with a wide range of applications.

## Data Availability

Data are available on reasonable demand.

## References

[1] Walker DR, Struk AM, Matsuki K (2016). How do deltoid muscle moment arms change after reverse total shoulder arthroplasty?. J Shoulder Elb Surg.

[2] Greiner SH, Back DA, Herrmann S (2010). Degenerative changes of the deltoid muscle have impact on clinical outcome after reversed total shoulder arthroplasty. Arch Orthop Trauma Surg.

[3] Wall B, Nové-Josserand L, O’Connor DP (2007). Reverse total shoulder arthroplasty: a review of results according to etiology. J Bone Jt Surg—Ser A.

[4] Ferraioli G, Tinelli C, Zicchetti M (2012). Reproducibility of real-time shear wave elastography in the evaluation of liver elasticity. Eur J Radiol.

[5] Itoh A, Ueno E, Tohno E (2006). Breast disease: Clinical application of US elastography for diagnosis. Radiology.

[6] Sebag F, Vaillant-Lombard J, Berbis J (2010). Shear wave elastography: A new ultrasound imaging mode for the differential diagnosis of benign and malignant thyroid nodules. J Clin Endocrinol Metab.

[7] Bhatia KSS, Rasalkar DD, Lee YP (2010). Real-time qualitative ultrasound elastography of miscellaneous non-nodal neck masses: applications and limitations. Ultrasound Med Biol.

[8] Brandenburg JE, Eby SF, Song P (2014). Ultrasound elastography: the new frontier in direct measurement of muscle stiffness. Arch Phys Med Rehabil.

[9] Ishikawa H, Muraki T, Sekiguchi Y (2015). Noninvasive assessment of the activity of the shoulder girdle muscles using ultrasound real-time tissue elastography. J Electromyogr Kinesiol.

[10] Ophir J, Céspedes I, Ponnekanti H (1991). Elastography: a quantitative method for imaging the elasticity of biological tissues. Ultrason Imaging.

[11] Schmalzl J, Fenwick A, Boehm D, Gilbert F (2017). The application of ultrasound elastography in the shoulder. J Shoulder Elb Surg.

[12] Sarvazyan APA, Rudenko OOV, Swanson SD (1998). Shear wave elasticity imaging: a new ultrasonic technology of medical diagnostics. Ultrasound Med Biol.

[13] Kot BCW, Zhang ZJ, Lee AWC (2012). Elastic modulus of muscle and tendon with shear wave ultrasound elastography: variations with different technical settings. PLoS ONE.

[14] Hatta T, Giambini H, Uehara K (2015). Quantitative assessment of rotator cuff muscle elasticity: reliability and feasibility of shear wave elastography. J Biomech.

[15] Muraki T, Ishikawa H, Morise S (2014). Ultrasound elastography-based assessment of the elasticity of the supraspinatus muscle and tendon during muscle contraction. J Shoulder Elb Surg.

[16] Takenaga T, Sugimoto K, Goto H (2015). Posterior shoulder capsules are thicker and stiffer in the throwing shoulders of healthy college baseball players. Am J Sports Med.

[17] Yuri T, Mura N, Yuki I (2018). Contractile property measurement of the torn supraspinatus muscle using real-time tissue elastography. J Shoulder Elb Surg.

[18] Hatta T, Giambini H, Sukegawa K (2016). Quantified mechanical properties of the deltoid muscle using the shear wave elastography: Potential implications for reverse shoulder arthroplasty. PLoS ONE.

[19] Muraki T, Ishikawa H, Morise S (2015). Ultrasound elastography–based assessment of the elasticity of the supraspinatus muscle and tendon during muscle contraction. J Shoulder Elb Surg.

[20] Faul F, Erdfelder E, Buchner A, Lang A-G (2009). Statistical power analyses using G*Power 3.1: tests for correlation and regression analyses. Behav Res Methods.

[21] Sarvazyan A, Rudenko O, Swanson S (1998) Shear wave elasticity imaging: a new ultrasonic technology of medical diagnostics. Ultrasound Med. … 10.1016/s0301-5629(98)00110-0 10385964

[22] Schmalzl J, Piepenbrink M, Buchner J (2020). Higher primary stability of tuberosity fixation in reverse fracture arthroplasty with 135° than with 155° humeral inclination. J shoulder Elb Surg.

[23] Kim K, Hwang HJ, Kim SG (2018). Can shoulder muscle activity be evaluated with ultrasound shear wave elastography?. Clin Orthop Relat Res.

[24] Roche CP, Diep P, Hamilton M (2013). Impact of inferior glenoid tilt, humeral retroversion, bone grafting, and design parameters on muscle length and deltoid wrapping in reverse shoulder arthroplasty. Bull Hosp Jt Dis.

[25] Schwartz DG, Kang SH, Lynch TS (2013). The anterior deltoid’s importance in reverse shoulder arthroplasty: A cadaveric biomechanical study. J Shoulder Elb Surg.

[26] Lädermann A, Williams MD, Melis B (2009). Objective evaluation of lengthening in reverse shoulder arthroplasty. J Shoulder Elb Surg.

[27] Romano AM, Braile A, Casillo P (2020). Onlay uncemented lateralized reverse shoulder arthroplasty for fracture sequelae type 1 with valgus/varus malunion: deltoid lengthening and outcomes. J Clin Med.

[28] Ho JC, Sabesan VJ, Iannotti JP (2013). Glenoid component retroversion is associated with osteolysis. J Bone Joint Surg Am.

